# Tunable and enhanced light emission in hybrid WS_2_-optical-fiber-nanowire structures

**DOI:** 10.1038/s41377-018-0115-9

**Published:** 2019-01-16

**Authors:** Jin-hui Chen, Jun Tan, Guang-xing Wu, Xue-jin Zhang, Fei Xu, Yan-qing Lu

**Affiliations:** 1 Key Laboratory of Intelligent Optical Sensing and Manipulation (Ministry of Education), College of Engineering and Applied Sciences, National Laboratory of Solid State Microstructures and Collaborative Innovation Center of Advanced Microstructures, Nanjing University, Nanjing, 210093 People’s Republic of China; 2 School of Physics, Nanjing University, Nanjing, 210093 People’s Republic of China

**Keywords:** Nonlinear optics, Optical properties and devices, Fibre optics and optical communications

## Abstract

In recent years, the two-dimensional (2D) transition metal dichalcogenides (TMDCs) have attracted renewed interest owing to their remarkable physical and chemical properties. Similar to that of graphene, the atomic thickness of TMDCs significantly limits their optoelectronic applications. In this study, we report a hybrid WS_2_-optical-fiber-nanowire (WOFN) structure for broadband enhancement of the light–matter interactions, i.e., light absorption, photoluminescence (PL) and second-harmonic generation (SHG), through evanescent field coupling. The interactions between the anisotropic light field of an optical fiber nanowire (OFN) and the anisotropic second-order susceptibility tensor of WS_2_ are systematically studied theoretically and experimentally. In particular, an efficient SHG in the WOFN appears to be 20 times larger than that in the same OFN before the WS_2_ integration under the same conditions. Moreover, we show that strain can efficiently manipulate the PL and SHG in the WOFN owing to the large configurability of the silica OFN. Our results demonstrate the potential applications of waveguide-coupled TMDCs structures for tunable high-performance photonic devices.

## Optical fiber nanowires: A tunable light source all wrapped up

Coating an optical fiber nanowire in an ultrathin film of tungsten sulfide enhances its optical properties for use in tiny photonic devices. Transition metal dichalcogenides are semi-conducting materials which, like graphene, demonstrate valuable and unique physical properties when they are thinned down to single molecular layers. Fei Xu and Yan-qing Lu at Nanjing University, China and co-workers achieved tunable, enhanced light emission from an optical fiber nanowire by wrapping it in an ultrathin layer of tungsten disulfide (WS_2_). They showed that second harmonic generation – the generation of higher-energy photons - was twenty times greater in their hybrid design than in a bare nanowire. Further, by placing strain on the wire, the researchers could manipulate photon-electron and photon-photon interactions in WS_2_, allowing control over optical properties including photoluminescence.

## Introduction

Layered transition metal dichalcogenides (TMDCs) have attracted significant renewed interest in recent years, from fundamental physics to applications, owing to advances in graphene research^1,2^. Although most of the TMDCs have been studied for decades, it has been recently revealed that atomically thin TMDCs can exhibit distinct properties compared with their bulk counterparts^2,3^. For example, MoS_2_ exhibits a transition from an indirect bandgap in the bulk to a direct bandgap in the monolayer owing to the lateral quantum confinement effect^4,5^. The reduced dielectric screening of the Coulomb interactions contributes to the extremely strong exciton effects^6,7^. The broken inversion symmetry and strong spin–orbit coupling in the monolayer lead to robust spintronics and valleytronics^8^, with a possibility for optical manipulation^9,10^. The lack of centrosymmetry in odd layers contributes to the giant second-order optical nonlinearity^11,12^. These pioneering studies indicate that TMDCs are promising candidates for electronic^13^, photonic^14–19^, and optoelectronic^20–23^ applications. Although the layered TMDCs exhibit considerably strong light–matter interactions in the visible/near-infrared spectrum owing to the exciton resonance effects^7,24,25^, an interaction enhancement is possible when considering the large discrepancy between the light wavelength and atomic thickness of the TMDCs, especially in the nonresonant spectrum region. The inherent flexibleness of two-dimensional (2D) TMDCs is advantageous for their integration to photonic structures, including optical waveguides^26–28^, microcavities^14–17,29–31^, and plasmonic structures^32–38^. Nevertheless, most of these hybrid structures do not utilize the tunable properties of the TMDCs, which can be easily manipulated by doping^6,23,39^, strain^40,41^, and other environmental effects^42^.

**Fig. 1:**
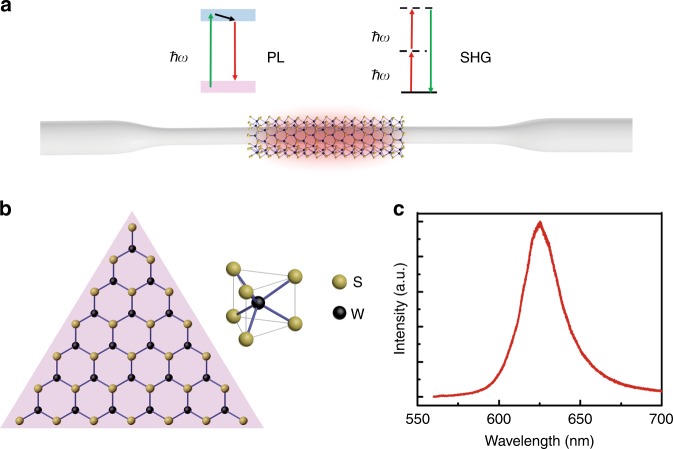
Design of the OFN integrated with a WS_2_ structure. **a** Schematic of the WOFN as a platform for enhancement of the PL and SHG in WS_2_. **b** Crystal structure of a triangular monocrystalline WS_2_, with a threefold symmetry. The right panel shows the prismatic cell of WS_2_. **c** PL spectrum of a WS_2_ monolayer film

In this study, we report a direct integration of monocrystalline monolayer WS_2_ to an optical fiber nanowire (WOFN) for broadband enhancement of light–matter interactions (Fig. 1a), i.e., photoluminescence (PL) and second-harmonic generations (SHGs). Through the evanescent field coupling effects in the optical fiber nanowire (OFN), the light–WS_2_ interaction length can be significantly extended^43,44^, which is free from the limitations of the atomic thickness in monolayer WS_2_. Moreover, the waveguide structure can also efficiently collect the light emission from the WS_2_ through the near-field coupling effect^27,45^. Although several previous studies have reported an integration of nanoflakes of TMDCs to an optical waveguide, most of them focused on the TMDCs’ extrinsic properties, such as saturation absorption effects in the infrared range for pulsed fiber lasers^46,47^, which can be attributed to defects in the TMDCs. Here, we experimentally demonstrate an in-waveguide tuning of the exciton wavelength of WS_2_ under a uniaxial strain^41^, owing to the large configurability and mechanical strength of the silica OFN^43,48^. In addition, we show that the SHG in the OFN can be significantly enhanced with the introduction of the WS_2_ layer, when considering its large second-order nonlinearity^49^. In the WOFN waveguide, the interactions between the anisotropic light field of the OFN and the anisotropic second-order susceptibility tensor of WS_2_ are carefully explored theoretically and experimentally. Furthermore, we reveal that the SHG in the WOFN can be controlled by the strain with a high sensitivity through the nonlinear multibeam interference effects. Our study can reveal a novel approach for tunable high-performance optical-waveguide-integrated linear and nonlinear devices.

## Results

The schematic of the WOFN is illustrated in Fig. 1a, which is achieved by laminating a piece of a WS_2_ monolayer in the waist region of an OFN using a modified microtransfer technique (Figs. S1 and  S2)^50^. The OFN is fabricated by flame brushing techniques, while the WS_2_ film is grown by chemical vapor deposition (CVD). Considering the typical grain size of the CVD-grown single-crystalline WS_2_, the effective encapsulating length of WS_2_ in the WOFN is usually within 100μm. The crystal structure of WS_2_ is illustrated in Fig. 1b, where two layers of sulfur atoms (S) are separated by one layer of tungsten (W) atoms; the W atoms exhibit a trigonal prismatic coordination. The PL spectrum of the transferred WS_2_ on a glass substrate indicates the direct bandgap characteristics (Fig. 1c)^41,51,52^.

**Fig. 2:**
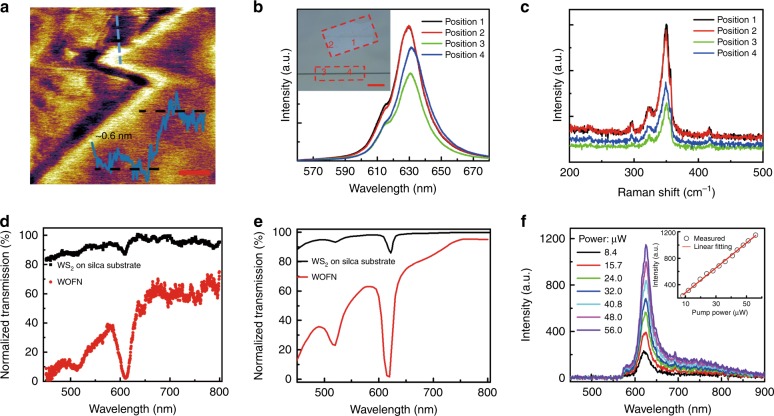
Characterizations of the WOFN structure. **a** Atomic force microscopy (AFM) image of a piece of WS_2_ on a sapphire substrate. The thickness of the WS_2_ film is ~0.6 nm, as shown in the inset. The scale-bar is 500 nm. **b** PL spectra of a transferred WS_2_ film on a glass slide and silica OFN; 1–4 denote the different positions labeled in the inset, at which the spectra are collected. The red dashed boxes outline the positions of the transferred WS_2_ on the WOFN and the residual WS_2_ film on the glass substrate. The scale-bar corresponds to 20 μm. **c** Raman spectra of the sample from the positions in **b**. **d** Measured normalized transmission spectra of the WOFN and WS_2_ on a silica substrate. The diameter of the OFN is ~800 nm, while the effective laminated length of WS_2_ on the WOFN is ~60 μm. **e** Calculated transmission spectra with the same structure parameters as those in **d**. **f** PL spectra of the WOFN with different pump power values (at 532 nm). The inset shows the linear relationship between the PL intensity and the pump light power

Atomic force microscopy (AFM) was used to determine the thickness of WS_2_ (Fig. 2a), which clearly indicated monolayer characteristics. To demonstrate the quality of the transferred WS_2_, we measured in situ the PL and Raman spectra using a continuous 532-nm excitation light source for the WOFN and WS_2_ on a glass substrate, as shown in Fig. 2b, c. The WOFN was put on a glass slide for measurement convenience. The inset in Fig. 2b shows four different positions, at which the optical spectra are collected. The PL spectrum peaks at approximately 630 nm, which corresponds to the *A*^-^ exciton (trion), which is the direct interband transition at the *K*-point in the hexagonal Brillouin zone. The shoulder peak of the PL at ~612 nm could be attributed to the neutral exciton *A*. We believe that the unintentional doping during the transfer process leads to the PL fingerprints of WS_2_^53^. The redshift of the *A*/*A*^-^ exciton of the WOFN (positions 3 and 4), compared with WS_2_ on the glass substrate (positions 1 and 2), most likely emerges owing to the geometrical curvature of the OFN and the residual strain introduced in the transfer process. With regard to the Raman fingerprint, for example, for position 1, five peaks are clearly resolved by the Lorentz fitting, at 296.4, 324.8, 349.3, 356.7, and 417.7 cm^–1^, which corresponds to different vibration modes of WS_2_^54^.

Figure 2d compares the measured absorption spectra of WS_2_ deposited on the end-face of a fiber patch cord and WOFN. The length of the integrated WS_2_ in the WOFN is ~60 μm. An optical-fiber-coupled halogen light source (SLS201/M, Thorlabs) is employed; the output spectra are analyzed using a fiber-coupled optical spectrometer (Fig. S3). Two prominent absorption peaks appeared at 610.2 nm (*A* exciton) and 510.6 nm (*B* exciton). The energy separation (~400 meV) between the *A* and *B* excitons is attributed to the energy splitting of the valence band owing to the spin–orbit coupling effect^24,52^. The magnitude of the exciton absorption in the WOFN (*A* exciton: ~97.7%) is significantly enhanced compared with the free-space illumination (*A* exciton: 13.0%), owing to the enhanced light–matter interactions in the WOFN. In addition, we employ the finite-element method to simulate the transmission spectrum of the WOFN (Fig. S4), as shown in Fig. 2e, which agrees well with the experimental results. The measured transmission loss in the infrared region is approximately 0.5 dB (Fig. S5), which is beneficial for nonlinear optics applications. Figure 2f shows the output PL spectra of the WOFN for different pump power values. The PL intensity exhibits an almost linear relationship with a pump power of up to 56 μW, as shown in the inset. We also conducted contrast experiments, and the results showed that the output PL intensity of the WOFN was higher than that of WS_2_ directly deposited on the optical fiber end-face. Moreover, the numerical simulation shows that the average one-directional coupling efficiency of the WS_2_ exciton emission to the OFN is ~12%, which attests to the superiority of the waveguide-coupled-WS_2_ structure for light excitation and collection (Fig. S7).

**Fig. 3:**
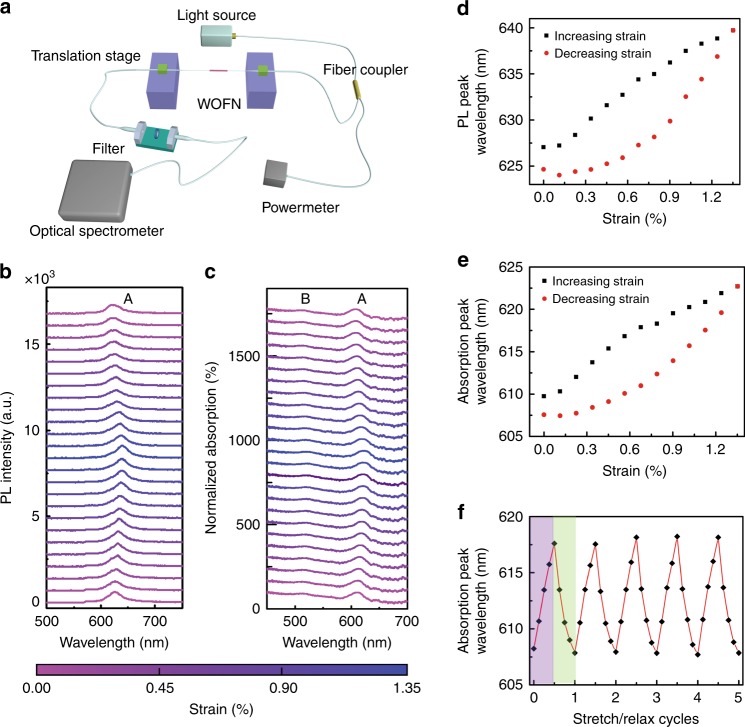
Strain manipulation of the PL and absorption spectra of the WOFN. **a** Experimental set-up for the in-waveguide tuning of the PL spectra of WS_2_ in the WOFN. The translation stage is used to apply a uniaxial strain on the WOFN. Variations of the **b** PL spectra and **c** absorption spectra of the WOFN with an increase and decrease in the strain. The curves from bottom to top correspond to the sequence of data acquisition. For clarity, the curves in **b** are vertically shifted by multiples of 700, while those in **c** are shifted by multiples of 70%. Variations of the **d** PL peak wavelength and **e** absorption peak wavelength in the A exciton region with the increase and decrease in the strain. **f** Dependence of the absorption peak wavelength of the WOFN during strain loading and unloading. The violet region corresponds to the increase in the strain, while the green region corresponds to the decrease in the strain. One cycle contains four steps of strain loading/unloading; each step corresponds to a strain of 0.22%

Strain engineering has been widely employed owing to the corresponding evolution of the electronic band structure of the 2D materials, including graphene and TMDCs^40,41,55,56^. Most studies employed the free-space coupling technique to detect the optical spectra of 2D materials as a function of the strain. This method is simple; however, miniaturization and integration are challenging. Figure 3a shows the experimental set-up for an in-line manipulation of the PL spectra of WS_2_. A uniaxial strain in the WOFN is applied by stretching using the translation stage; the strain is transferred to the attached WS_2_ film. The WOFN was illuminated using an excitation light source (~ 40 μW, 532 nm); the output PL was analyzed using an optical-fiber-coupled spectrometer. Unless otherwise stated, the WOFN sample under the strain manipulation is the same as that presented in Fig. 2d, the diameter of which is 795 ± 6 nm (Fig. S8); the strain values are calculated using the ratio of the elongated length of the WOFN to its original length.

Figure 3b, c summarize the PL and absorption spectra of the WOFN as a function of the strain, which was increased from 0% to 1.35% and then decreased to 0% (from bottom to top). The emission spectra exhibit a prominent redshift with the increase in the strain; the corresponding absorption spectra exhibit similar patterns. A linear fitting shows that the slope of the PL peak wavelength with respect to the strain is 10.1 nm/% strain (–30 meV/% strain) during the increase in the strain, which is comparable to the values reported in other studies^40,41,57,58^. The tuning range of the exciton wavelength in TMDCs is mainly limited by the direct-indirect bandgap transition induced by certain strain magnitude. Both the emission and absorption spectra are not completely reversible. Quasi “hysteresis” loops of the PL peak wavelength and absorption peak wavelength are observed in Fig. 3d, e. Further, we measured the peak wavelength of absorption during a strain loop test, as shown in Fig. 3f. The spectral response is almost recovered after one cycle, even though there is a hysteresis. The hysteresis of the WOFN could be attributed to the interface relaxation effect in WS_2_–silica; further studies are required to elucidate the origin of this phenomenon. A possible solution to the hysteresis problem is to coat a thin layer of a low-refractive-index elastomer (polydimethylsiloxane (PDMS)) on the surface of the WOFN, which can help to fasten WS_2_ on the substrate;^57^ however, the waveguide dispersion can be significantly modulated. Although the waveguide dispersion has a small effect on the PL, it can significantly influence the nonlinear optical phenomena in the WOFN, as discussed in the next section. It should be noted that for practical applications, the WOFN should be well encapsulated to enhance the robustness and long-term stability^59^.

Monolayer TMDCs exhibit a large second-order nonlinearity (*χ*^(2)^) owing to the breaking of the inversion symmetry; *χ*^(2)^ can be further enhanced in the exciton resonant region^11,12,49^. Most of the previous studies reported an SHG in the TMDCs when using the free-space coupling technique with a low conversion efficiency, which is limited by the small light–matter-interaction cross section. An intuitive method to improve the SHG conversion is to employ the optical waveguide coupling techniques. In contrast to the direct illumination method, a phase matching is needed for a high conversion efficiency in waveguides. In a fused silica fiber, the value of *χ*^(2)^ in the bulk is low, while that at the surface is considerable owing to the symmetry breaking at the air–silica interface. To characterize the enhancement of the SHG in the WOFN, we compared the SHG in an OFN before and after the transfer of WS_2_.

**Fig. 4:**
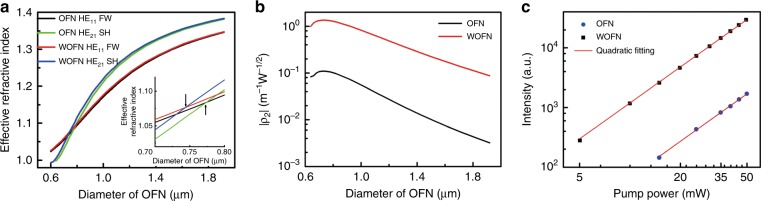
SHG in the WOFN. **a** Effective mode index of the bare OFN and WOFN as a function of the OFN diameter at the FW at 1550 nm and SH at 775 nm. The FW with a mode index HE_11_ and SH with a mode index HE_21_ are considered. The inset shows an enlarged view at the phase-matching region. **b** Nonlinear coupling parameter |*ρ*_2_| as a function of the OFN diameter for the OFN and WOFN structures (FW at 1550 nm). **c** SHG intensity dependence (on a log–log scale) on the pump power for the OFN and WOFN. The SHG intensities are measured for the same OFN before and after the deposition of WS_2_

Figure 4a shows the OFN/WOFN dispersions as a function of the diameter of the waveguide for the fundamental wave (FW) at 1550 nm and second-harmonic (SH) at 775 nm. The waveguide dispersion is slightly modified upon the introduction of the WS_2_ layer. In particular, the phase-matching point is shifted (in terms of the OFN diameter) by ~30 nm, as shown in the inset of Fig. 4a. Although there are other optical modes, such as HE_11_-(FW)-TM_01_-(SH) of the WOFN, that satisfy the phase-matching conditions, the symmetry of the second-order nonlinearity tensor of WS_2_^11,12,49^ inhibits the harmonic generation (Supplementary Note 2.2). By solving the coupling-wave equation in the small signal approximation, we can find that the SHG intensity (*P*_SHG_) can be derived as follows:

1 $$P_{{\mathrm{SHG}}} = P_{{\mathrm{FW}}}^2|{\mathrm{\rho }}_2|^2{\mathrm{L}}^2\left[ {\frac{{{\mathrm{sin}}(\Delta {\mathrm{\beta L/}}2)}}{{\Delta {\mathrm{\beta L/}}2}}} \right]^2$$

where *P*_FW_ is the pump power of FW, *ρ*_2_ is the nonlinear coupling parameter, *L* is the effective interaction length along the waveguide, and Δ*β* = 2*β*_FW_ – *β*_SHG_ is the phase mismatch between the fundamental and second-harmonic waves. The nonlinear coupling parameter *ρ*_2_ is defined as the overlap integral:^60^

2 $$\begin{array}{l}{\mathrm{\rho }}_{\mathrm{2}} = \frac{{{\mathrm{\omega }}_{\mathrm{2}}}}{{4N_1\sqrt {N_2} }}{\int} {{\mathop{\rm e}\nolimits} _2{\boldsymbol \cdot {P}}^{(2)}dS} \\ N_j = \frac{1}{2}{\int} {{\mathrm{|}}{\mathop{\rm e}\nolimits} _j} ^ \ast \times {\mathop{\rm h}\nolimits} _j \cdot {\mathop{\rm z}\nolimits} |dS\,\,\,\,\,\,\,\,\,\,\,\,\,j = 1,2\end{array}$$

where *ω*_2_ is the second-harmonic frequency, and *N*_1_ and *N*_2_ are the normalized field factors for FW and SH, respectively. *P* ^(2)^ is the second-order nonlinear polarization, which can be calculated according to the second-order susceptibility tensor of the materials (Supplementary Note 2.2). Figure 4b compares the nonlinear coupling parameters |*ρ*_2_| of the OFN and WOFN, as a function of the waveguide diameter. The values of |*ρ*_2_| of the WOFN are one order of magnitude larger than those of the OFN, which implies that the power conversion efficiency of the WOFN is two orders of magnitude larger than that of the OFN under the same conditions. As the physical interpretation of *ρ*_2_ is attributed to the overlap integral of the optical mode of the FW and SH^60^, |*ρ*_2_| initially increased with the decrease in the waveguide diameter, and then decreased after the matching point. The crystal orientation alignment in the WOFN has a slight influence on |*ρ*_2_| (Fig. S9). The quadratic dependences of the output SHGs in the OFN and WOFN on the pump power are clearly demonstrated in Fig. 4c. The SHG intensity of the WOFN is approximately 20 times larger than that of the OFN, which is comparable to the theoretical value considering the insertion loss and imperfect transfer of WS_2_ (Fig. S11). In addition, we pumped a sample with WS_2_ directly deposited on the surface of a cleaved optical fiber, and no SHG was detected for input powers of up to 60 mW. Intuitively, the waveguide enhancement of SHG compared to the free-space coupling will be proportional to the effective interaction length square if the phase matching conditions are satisfied and the additional insertion loss is neglected.

**Fig. 5:**
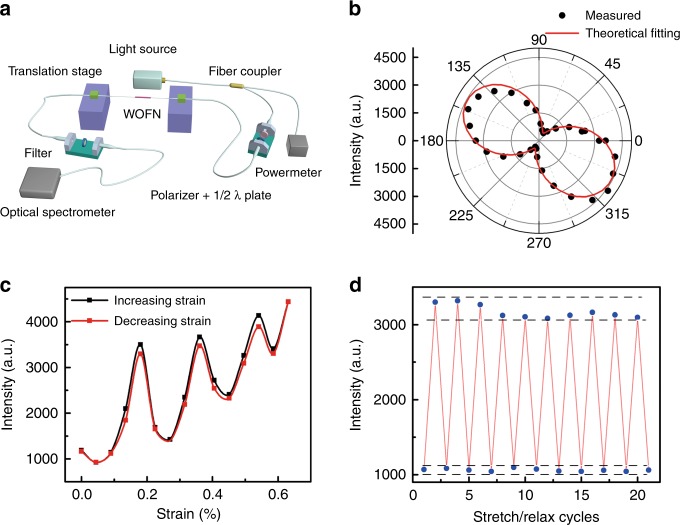
Strain manipulation of the SHG in the WOFN. **a** Experimental set-up for control of the SHG in the WOFN. The WOFN is clamped on two translation stages. The linear polarization of the pump light can be tuned by a composite polarizer and half-wave plate. **b** Polar image of the measured SHG intensity, as a function of the linear polarization of the pump light with a fixed light power. **c** Reversible strain modulation of the SHG intensity of the WOFN. **d** Cycling tests of strain modulation of the SHG of the WOFN. Each stretch/relax step corresponds to a strain value of 0.45%. The dashed lines indicate the intensity fluctuations of the SHG

To investigate the possible effects on the SHG in the WOFN, we set-up an experimental configuration, as shown in Fig. 5a. The output SHG intensity depends on the linear polarization of the pump FW, as shown in Fig. 5b. The SHG intensity should be independent of the polarization of the FW owing to the circular symmetry of the WOFN, assuming a perfect WS_2_ encapsulation. Nevertheless, incomplete coverage of WS_2_ on the WOFN is always present owing to the transfer technique, which leads to the polarization extinction. A theoretical fitting reveals that the WS_2_ coverage ratio is ~75%. The polarization extinction spectrum of the WOFN can serve as a guide to characterize the WS_2_ transfer quality (Fig. S10). It is intuitive that thinner poly(methyl methacrylate) (PMMA) film leads to a higher WS_2_ coverage ratio, while the strength of the film will be compromised, which is challenging for the transfer process. Figure 5c shows the SHG intensity as a function of the applied strain; the oscillations are clearly resolved. The modulation process is almost reversible, as shown in Fig. 5d. The SHG intensity fluctuations are within 7%, most likely owing to the instability of the pump power and measurement configuration. As the measured SHGs are far away from the exciton resonant region of WS_2_, we conclude that this modulation is most likely not caused by the change of *χ*^(2)^, but attributed to the nonlinear interference between the harmonic waves generated at different parts of the WOFN, i.e., at positions with and without a WS_2_ deposition (Figs. S13 and  S14). Although the modulation strategy here is less reproducible in the OFN platform experimentally, theoretically, if we can well control the geometry of the WOFN, the output SHG can be well predicted. Furthermore, this method can be readily employed in flexible on-chip devices, in which the configuration is highly reproducible.

## Discussion

In summary, we demonstrated a hybrid optical fiber waveguide integrated with a WS_2_ monolayer for the enhancement of the PL and SHG through evanescent field coupling. We revealed that the in-line strain can efficiently manipulate the photon–electron and photon–photon interactions in WS_2_. The waveguide-coupled PL spectra and exciton absorptions of WS_2_ were experimentally linearly tuned over a wavelength range of 10 nm. Moreover, we systematically analyzed the harmonic generation in the WOFN structure and showed that the SHG in the WOFN was more than one order of magnitude larger than that in the bare OFN under the same conditions. This value can be further enhanced if the pump light is tuned at the exciton resonance or peak joint-density-of-states regions (Fig. S12)^49^. Nevertheless, the waveguide matching conditions imply that a shorter-wavelength pump light source requires a thinner OFN waveguide structure^60^, which is very challenging to be experimentally achieved (Fig. S6). This unique platform can have broad applications in optical fiber sensing and nonlinear optics. For optical fiber sensing, this kind of sensor can operate in a passive light absorption mode or active light emission mode depending on the optical measurement system. Compared to the traditional optical nanofiber/microfiber sensors based on external resonating structures^43,61^, this hybrid sensor is based on the electronic band structure of WS_2_ and its response to the loaded strain^40,41^, which will be robust to environmental perturbations. For the nonlinear optics, we experimentally and theoretically show that the SHG in the hybrid waveguide can be dynamically tuned with the strain, which can be attributed to the nonlinear interference effects. Another possible application is to integrate this device to an active fiber laser circuit for tunable pulsed light generations^62,63^, in which the WS_2_ might serve as tunable saturable absorbers. We believe that our structure design can be easily applied to other TMDCs, which can pave the way for the design of tunable waveguide-coupled light sources.

## Materials and methods

### Material and device characterizations

The surface morphology of WS_2_ (6Carbon Technology, Shenzhen) on the sapphire substrate was measured using AFM (Cypher ES Polymer Edition, Asylum Research). The Raman and PL spectra of WS_2_ on the flat substrate were recorded at room temperature in air using a LabRam HR 800 Evolution system (HORIBA Jobin Yvon) with an excitation line of 532 nm. We used gratings with 1800 gr/mm and 600 gr/mm for the Raman measurement and PL characterization, respectively. The Raman band of Si at 520 cm^−1^ was used as a reference to calibrate the spectrometer. The absorption and PL spectra of the WOFN device were measured using two optical-fiber-coupled spectrometers, USB2000+ (~0.4 nm resolution, Ocean Optics) and NOVA (~0.8 nm resolution, Idea Optics Co., China). A filtered nanosecond pulsed fiber laser (pulse width: 10 ns, repetition frequency: 1 MHz, APFL-1550-B-CUSTOM, SPL Photonics Co., Ltd) was used to pump the WOFN for the SHG characterizations; the output signal was filtered and analyzed using a fiber-coupled spectrometer.

### Strain response measurement

The WOFN was clamped on two translation stages; the in-line stretching of the WOFN was generated by the linear motor stage (XML, Newport). As the applied strain in the WOFN is highly nonuniform, we calibrated the strain value of the waist region by modeling the geometry of the WOFN.

## Supplementary information

revised-supporting information

## References

[1] Butler SZ (2013). Progress, challenges, and opportunities in two-dimensional materials beyond graphene. ACS Nano.

[2] Wang QH, Kalantar-Zadeh K, Kis A, Coleman JN, Strano MS (2012). Electronics and optoelectronics of two-dimensional transition metal dichalcogenides. Nat. Nanotechnol..

[3] Mak KF, Shan J (2016). Photonics and optoelectronics of 2D semiconductor transition metal dichalcogenides. Nat. Photonics.

[4] Splendiani A (2010). Emerging photoluminescence in monolayer MoS_2_. Nano Lett..

[5] Mak KF, Lee CG, Hone J, Shan J, Heinz TF (2010). Atomically thin MoS_2_: a new direct-gap semiconductor. Phys. Rev. Lett..

[6] Mak KF (2013). Tightly bound trions in monolayer MoS_2_. Nat. Mater..

[7] Ramasubramaniam A (2012). Large excitonic effects in monolayers of molybdenum and tungsten dichalcogenides. Phys. Rev. B.

[8] Xiao D, Liu GB, Feng WX, Xu XD, Yao W (2012). Coupled spin and valley physics in monolayers of MoS_2_ and other group-VI dichalcogenides. Phys. Rev. Lett..

[9] Zeng HL, Dai JF, Yao W, Xiao D, Cui XD (2012). Valley polarization in MoS_2_ monolayers by optical pumping. Nat. Nanotechnol..

[10] Mak KF, He KL, Shan J, Heinz TF (2012). Control of valley polarization in monolayer MoS_2_ by optical helicity. Nat. Nanotechnol..

[11] Malard LM, Alencar TV, Barboza APM, Mak KF, de Paula AM (2013). Observation of intense second harmonic generation from MoS_2_ atomic crystals. Phys. Rev. B.

[12] Kumar N (2013). Second harmonic microscopy of monolayer MoS_2_. Phys. Rev. B.

[13] Radisavljevic B, Radenovic A, Brivio J, Giacometti V, Kis A (2011). Single-layer MoS_2_ transistors. Nat. Nanotechnol..

[14] Gan XT (2013). Controlling the spontaneous emission rate of monolayer MoS_2_ in a photonic crystal nanocavity. Appl. Phys. Lett..

[15] Wu SF (2015). Monolayer semiconductor nanocavity lasers with ultralow thresholds. Nature.

[16] Ye Y (2015). Monolayer excitonic laser. Nat. Photonics.

[17] Liu XZ (2014). Strong light–matter coupling in two-dimensional atomic crystals. Nat. Photonics.

[18] Koperski M (2015). Single photon emitters in exfoliated WSe_2_ structures. Nat. Nanotechnol..

[19] He YM (2015). Single quantum emitters in monolayer semiconductors. Nat. Nanotechnol..

[20] Yin ZY (2012). Single-layer MoS_2_ phototransistors. ACS Nano.

[21] Sundaram RS (2013). Electroluminescence in single layer MoS_2_. Nano Lett..

[22] Ross JS (2014). Electrically tunable excitonic light-emitting diodes based on monolayer WSe_2_ p-n junctions. Nat. Nanotechnol..

[23] Zhang YJ, Oka T, Suzuki R, Ye JT, Iwasa Y (2014). Electrically switchable chiral light-emitting transistor. Science.

[24] Li YL (2014). Measurement of the optical dielectric function of monolayer transition-metal dichalcogenides: MoS_2_, MoSe_2_, WS_2_, and WSe_2_. Phys. Rev. B.

[25] Liu HL (2014). Optical properties of monolayer transition metal dichalcogenides probed by spectroscopic ellipsometry. Appl. Phys. Lett..

[26] Chen HT (2017). Enhanced second-harmonic generation from two-dimensional MoSe_2_ on a silicon waveguide. Light.

[27] Schell AW, Takashima H, Tran TT, Aharonovich I, Takeuchi S (2017). Coupling quantum emitters in 2D materials with tapered fibers. ACS Photonics.

[28] Tonndorf P (2017). On-chip waveguide coupling of a layered semiconductor single-photon source. Nano Lett..

[29] Gan XT (2018). Microwatts continuous-wave pumped second harmonic generation in few- and mono-layer GaSe. Light.

[30] Fang L (2018). Multiple optical frequency conversions in few-layer GaSe assisted by a photonic crystal cavity. Adv. Opt. Mater..

[31] Li YZ (2017). Room-temperature continuous-wave lasing from monolayer molybdenum ditelluride integrated with a silicon nanobeam cavity. Nat. Nanotechnol..

[32] Wang Z (2016). Giant photoluminescence enhancement in tungsten-diselenide-gold plasmonic hybrid structures. Nat. Commun..

[33] Najmaei S (2014). Plasmonic pumping of excitonic photoluminescence in hybrid MoS_2_-Au nanostructures. ACS Nano.

[34] Chen HT (2016). Manipulation of photoluminescence of two-dimensional MoSe_2_ by gold nanoantennas. Sci. Rep..

[35] Gong SH, Alpeggiani F, Sciacca B, Garnett EC, Kuipers L (2018). Nanoscale chiral valley-photon interface through optical spin-orbit coupling. Science.

[36] Kang YM (2014). Plasmonic hot electron induced structural phase transition in a MoS_2_ monolayer. Adv. Mater..

[37] Akselrod GM (2015). Leveraging nanocavity harmonics for control of optical processes in 2D semiconductors. Nano Lett..

[38] Zheng D (2017). Manipulating coherent plasmon–exciton interaction in a single silver nanorod on monolayer WSe_2_. Nano Lett..

[39] Seyler KL (2015). Electrical control of second-harmonic generation in a WSe_2_ monolayer transistor. Nat. Nanotechnol..

[40] He KL, Poole C, Mak KF, Shan J (2013). Experimental demonstration of continuous electronic structure tuning via strain in atomically thin MoS_2_. Nano Lett..

[41] Wang YL (2015). Strain-induced direct–indirect bandgap transition and phonon modulation in monolayer WS_2_. Nano Res..

[42] Huang YX, Guo JH, Kang YJ, Ai Y, Li CM (2015). Two dimensional atomically thin MoS_2_ nanosheets and their sensing applications. Nanoscale.

[43] Brambilla G (2009). Optical fiber nanowires and microwires: Fabrication and applications. Adv. Opt. Photonics.

[44] Kou JL, Chen JH, Chen Y, Xu F, Lu YQ (2014). Platform for enhanced light-graphene interaction length and miniaturizing fiber stereo devices. Optica.

[45] Yalla R, Le Kien F, Morinaga M, Hakuta K (2012). Efficient channeling of fluorescence photons from single quantum dots into guided modes of optical nanofiber. Phys. Rev. Lett..

[46] Yan PG (2015). Microfiber-based WS_2_-film saturable absorber for ultra-fast photonics. Opt. Mater. Express.

[47] Du J (2014). Ytterbium-doped fiber laser passively mode locked by few-layer Molybdenum disulfide (MoS_2_) saturable absorber functioned with evanescent field interaction. Sci. Rep..

[48] Tong LM (2003). Subwavelength-diameter silica wires for low-loss optical wave guiding. Nature.

[49] Janisch C (2014). Extraordinary second harmonic generation in tungsten disulfide monolayers. Sci. Rep..

[50] Wu XQ (2016). Effective transfer of micron-size graphene to microfibers for photonic applications. Carbon N. Y..

[51] Zhao WJ (2013). Evolution of electronic structure in atomically thin sheets of WS_2_ and WSe_2_. ACS Nano.

[52] Zhu BR, Chen X, Cui XD (2015). Exciton binding energy of monolayer WS_2_. Sci. Rep..

[53] Cong CX, Shang JZ, Wang YL, Yu T (2018). Optical properties of 2D semiconductor WS_2_. Adv. Opt. Mater..

[54] Berkdemir A (2013). Identification of individual and few layers of WS_2_ using Raman spectroscopy. Sci. Rep..

[55] Ni ZH (2008). Uniaxial strain on graphene: Raman spectroscopy study and band-gap opening. ACS Nano.

[56] Naumis GG, Barraza-Lopez S, Oliva-Leyva M, Terrones H (2017). Electronic and optical properties of strained graphene and other strained 2D materials: a review. Rep. Prog. Phys..

[57] Schmidt R (2016). Reversible uniaxial strain tuning in atomically thin WSe_2_. 2D Mater..

[58] He X (2016). Strain engineering in monolayer WS_2_, MoS_2_, and the WS_2_/MoS_2_ heterostructure. Appl. Phys. Lett..

[59] Li, J. H., Chen, J. H., & Xu, F. Sensitive and wearable optical microfiber sensor for human health monitoring. *Adv. Mater. Technol.*10.1002/admt.201800296.

[60] Lægsgaard J (2010). Theory of surface second-harmonic generation in silica nanowires. J. Opt. Soc. Am. B.

[61] Tong LM (2018). Micro/nanofibre optical sensors: challenges and prospects. Sensors.

[62] Chen JH (2015). Microfiber-coupler-assisted control of wavelength tuning for Q-switched fiber laser with few-layer molybdenum disulfide nanoplates. Opt. Lett..

[63] Qin CB, Gao Y, Qiao ZX, Xiao LT, Jia ST (2016). Atomic-layered MoS_2_ as a tunable optical platform. Adv. Opt. Mater..

