# Supplementary material for: Tunable and enhanced light emission in hybrid WS2-optical-fiber-nanowire structures
Source: Light Sci Appl. 2019 Jan 16;8:8. doi: 10.1038/s41377-018-0115-9 (PMC6333622; doi:10.1038/s41377-018-0115-9)
Supplement: Supplementary file 1 — revised-supporting information [file 41377_2018_115_MOESM1_ESM.docx]

**Supplementary Information**

**Tunable and enhanced light emission in hybrid WS_2_-optical-fiber-nanowire structures**

Jin-hui Chen^1^, Jun Tan^2^, Guang-xing Wu^1^, Xue-jin Zhang^1^, Fei Xu^1,*^, and Yan-qing Lu^1,*^

*feixu@nju.edu.cn and yqlu@nju.edu.cn

^1^ Key Laboratory of Intelligent Optical Sensing and Manipulation (Ministry of Education)，College of Engineering and Applied Sciences, National Laboratory of Solid State Microstructures and Collaborative Innovation Center of Advanced Microstructures, Nanjing University, Nanjing 210093, P. R. China

^2^School of Physics, Nanjing University, Nanjing 210093, P. R. China

**Supplementary Note 1: Sequential fabrication of the WS_2_–optical-fiber-nanowire (OFN) hybrid structure**

**
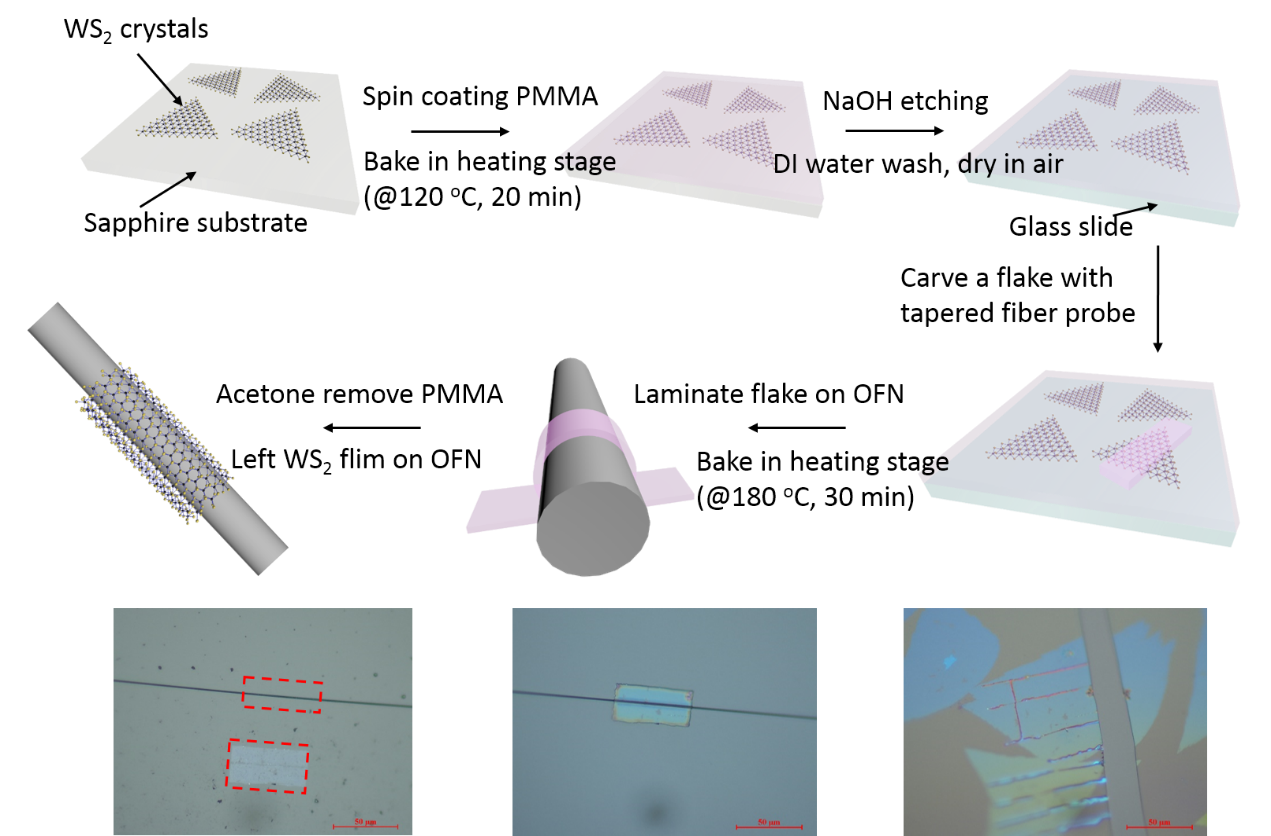
**

**Fig. S1** **Sequential fabrication of the WOFN.** The optical microscopy images correspond to the intermediate process. The red dashed boxes indicate the transferred WS_2_ on the WOFN and glass slide. The scale-bar corresponds to 50 μm

A monolayer monocrystalline WS_2_ was spin coated on a sapphire substrate with a 4 wt% poly(methyl methacrylate) (PMMA)–anisole solution, and heated on a hot plate at 120 °C for 20 min. The sample was then immersed in a 3 M NaOH solution for 4 h. This process partially etched the sapphire substrate and released a PMMA–WS_2_ film, which was transferred to deionized water to remove the NaOH residuals, and then transferred onto a clean glass slide and subsequently dried in air for several hours. While the CVD grown single crystal WS_2_ are separated from each other, the spin-coated PMMA can help visualize the regular shape of each flake, which might be attributed to the optical interference and absorption/reflection contrast of WS_2_. The obtained PMMA–WS_2_ was then cut into strips with a tapered probe under an optical microscope. The cutting direction was usually along the triangle perpendicular of WS_2_ film. Through the micro-transfer technique, we can make sure that the transferred PMMA film is fully filled with WS_2_ flake. An OFN was then drawn by flame-brushing techniques with a diameter of ~ 0.8 μm. The OFN was fixed on a clean glass substrate, and a PMMA-WS_2_ strip was aligned on the surface of the OFN, which was then heated on a hot plate for 30 min at 180 °C. The encapsulated OFN was washed with acetone to remove the protective PMMA film; WS_2_ remained on the OFN surface (Figure S2). The sequential fabrication process is shown in Fig. S1.


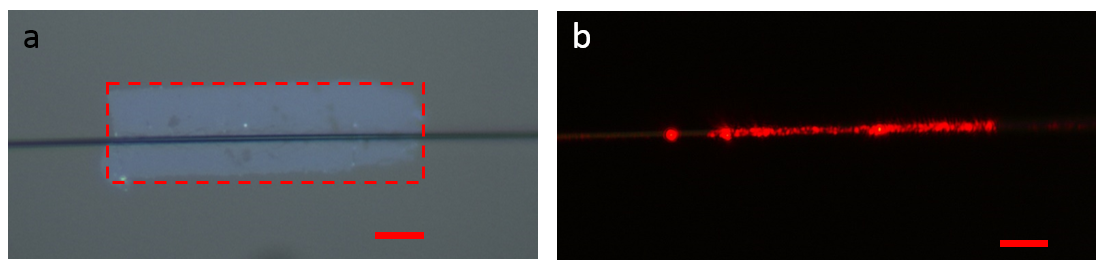


**Fig. S2** **Optical microscopy image of a typical WOFN.** **a** Bright-field image of the WOFN. **b** Dark-field image of the WOFN, illuminated with a red light source. The scattering field was induced by the encapsulated WS_2_ film. The scale-bar corresponds to 10 μm

**Supplementary Note 2: Modelling of the absorption and second-harmonic generation (SHG) in the WOFN**

**2.1** **Modelling of the WS_2_ monolayer film**

The dielectric function of WS_2_ is according to Refs. ^1, 2^. The surface conductivity of WS_2_ can be calculated as:

$\sigma^{s}=j\omega\varepsilon_{0}d(\varepsilon_{r}-1)$ (1)

where *ε* _0_ is the electric permittivity of vacuum, *d* is the thickness of the WS_2_ monolayer, and *ε _r_* is the complex permittivity of WS_2_.

For WS_2_ on a silica substrate, the transmission is:

$T=\frac{4n_{1}n_{2}}{(n_{1}+n_{2}+\sqrt{\frac{\mu_{0}}{\varepsilon_{0}}}\sigma^{s})^{2}}$ (2)

where *n*_1_ and *n*_2_ are the refractive indices of the environment (air) and substrate (silica).


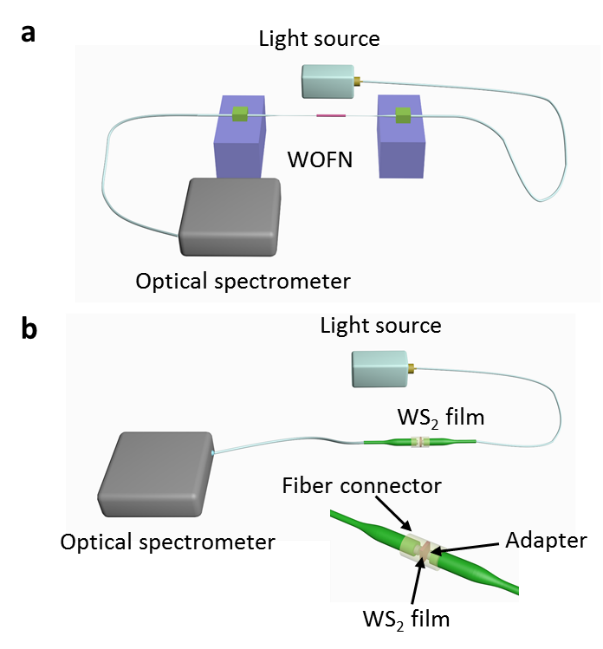


**Fig. S3 Schematic of the optical configurations for measuring the absorption spectra of (a) WOFN, and (b) WS_2_ flake on optical fiber end-face**





**Fig. S4** **Calculated waveguide dispersions of the OFN and WOFN structures.** The diameters of the OFN and WOFN are 0.8 μm. In the numerical modeling, WS_2_ is set as a dielectric layer with a thickness of 0.62 nm; the optical dispersion of WS_2_ is based on the results in Ref. ^1^. The dispersion of the silica material is also considered ^3^. The peak wavelengths of the imaginary part of the effective index for the WOFN correspond to the exciton absorption regions





**Fig. S5** **Measured transmission spectra of the OFN and WOFN.** The transmission spectra of an OFN are measured before and after the encapsulation with a layer of WS_2_. The diameter of the OFN is ~ 0.8 μm, while the effective laminated length of WS_2_ on the WOFN is ~ 60 μm





**Fig. S6** **Relation between phase matched SHG wavelength and OFN diameter, where the fundamental frequency mode is HE_11_ and second harmonic mode is HE_21_**


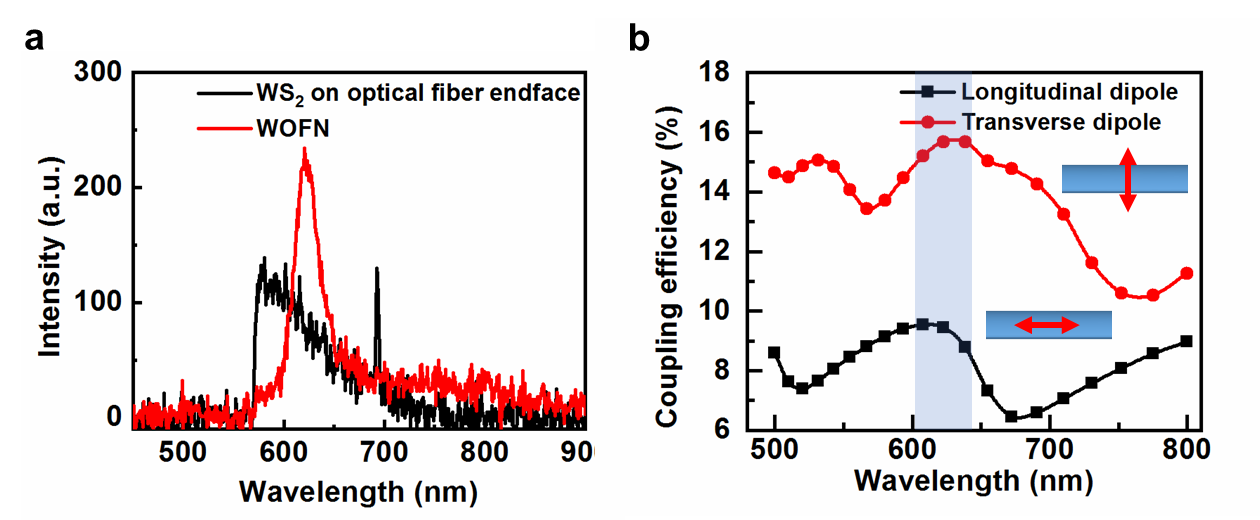


**Fig. S7** (**a**) Measured PL spectra of WS_2_. We compare the PL spectra of WS_2_ deposited on an optical fiber end-face (WOFE) and WOFN. The excitation (at 532 nm) and collection of the spectra are performed with an all-in optical fiber circuit. The PL of the WOFE is covered by the background signal, which could be attributed to the non-elastic scattering at the silica fiber or protective polymer coating of the fiber. The narrow peak emission around 693 nm is probably from the non-elastic scattering signal in the U-bench (FBP-A-FC, Thorlabs) that we employed for pump light filtering. In contrast, the PL is clearly resolved in the WOFN despite the high insertion loss in the visible band. (**b**) Directional coupling efficiency of WS_2_ exciton to OFN. To quantitatively calculate the coupling efficiency, three dimensional finite-difference time-domain (FDTD) simulations were performed on a commercial package (FDTD Solution, Lumerical). The PL emission from WS_2_ is modeled as dipoles with polarization along the surface of an OFN ^4^ and the diameter of the microfiber waveguide is 800 nm corresponding to the fabricated samples. The blue stripe indicates the monolayer WS_2_ PL spectrum region. Inset shows the dipoles with different polarization directions on the OFN


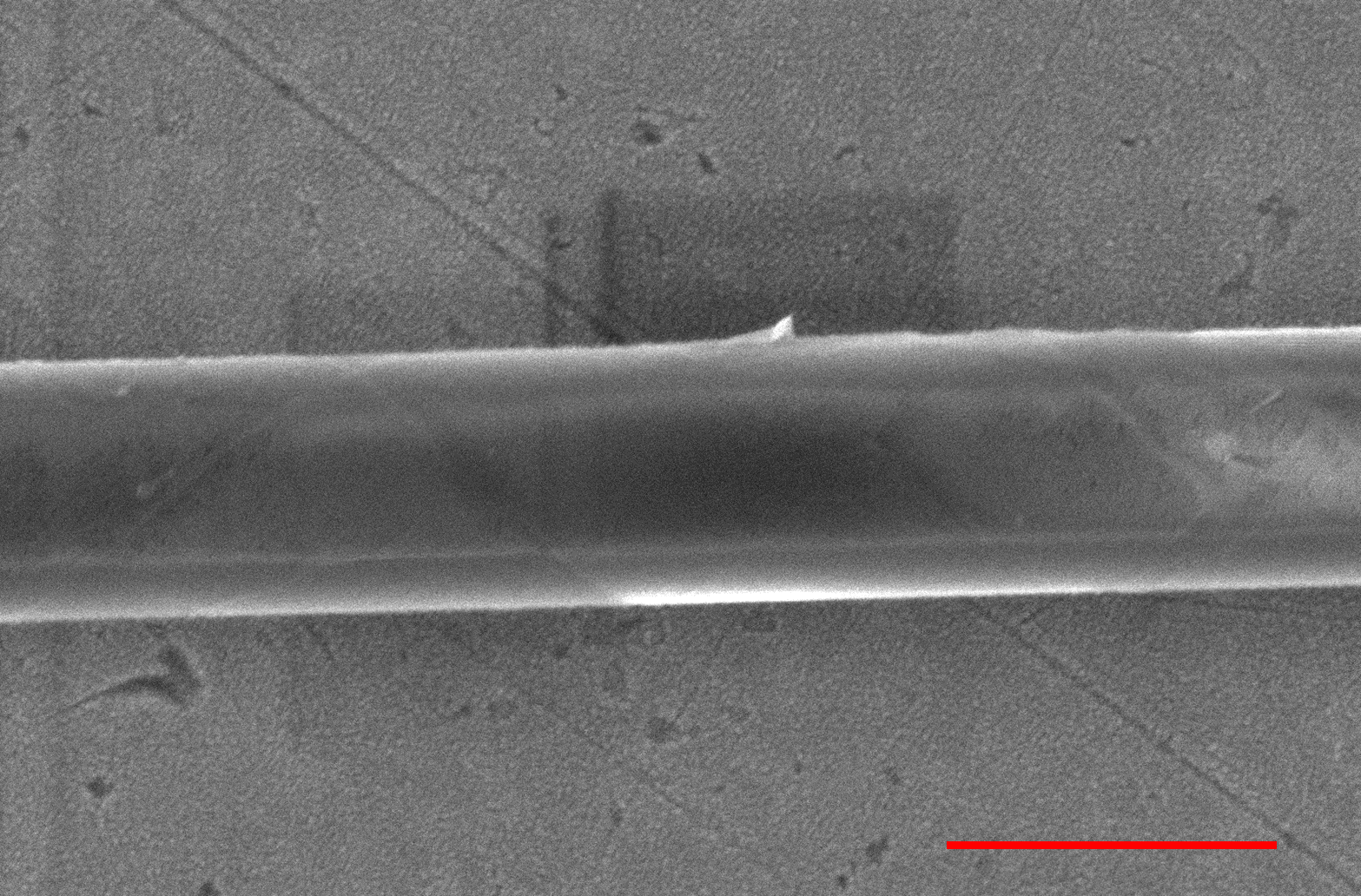


**Fig. S8 SEM image of a WOFN.** The scale bar is 1 μm

**2.2** **Modelling of the WOFN for the SHG**

In the small-signal limit, where the pump depletion is negligible, the SHG can be described by the equation ^5^:

$\frac{dA_{2}}{dz}=i\rho_{2}A_{1}^{2}\exp(i\Delta\beta z)-\alpha A_{2}$ (3)

where *A*_1_ and *A*_2_ are the field amplitudes of the fundamental and second-harmonic signals, respectively, *Δβ=2β*_1_*-β*_2_ is the phase mismatch between the fundamental and second-harmonic waves, *α* is the optical loss, and *ρ*_2_ is the overlap integral:

$\text{ρ}_{2}=\frac{\text{ω}_{2}}{4N_{1}\sqrt{N_{2}}}\int\mathop{\mathrm{e}_{2}}\limits\cdot\mathop{\mathrm{P}^{\text{(2)}}}\limits dS$ (4)

$N_{j}=\frac{1}{2}\int\text{|}\mathrm{e}_{j}^{*}\times\mathrm{h}_{j}\cdot\mathrm{z}|dS\begin{matrix} & \end{matrix}j=1,2$ (5)

The electric and magnetic fields of the guided modes are expressed as:

$\begin{matrix} \mathrm{E}\text{(}\mathrm{r}\text{, }\omega_{j}\text{)}=A_{j}\text{(}\omega_{j}\text{)}\mathrm{e}_{j}\text{(}\mathrm{r}\text{, }\omega_{j}\text{)exp(i(}\beta z\text{-}\omega_{j}t\text{))} \\ \mathrm{H}\text{(}\mathrm{r}\text{, }\omega_{j}\text{)}=A_{j}\text{(}\omega_{j}\text{)}\mathrm{h}_{j}\text{(}\mathrm{r}\text{, }\omega_{j}\text{)exp(i(}\beta z\text{-}\omega_{j}t\text{))} \end{matrix}$ (6)

***P*** ^(2)^ is the second-order nonlinear polarization:

$\mathrm{P}^{\text{(2)}}\text{(}\mathrm{r}\text{)}\approx\mathrm{P}_{s}^{\text{(2)}}\text{(}\mathrm{r}\text{)}=\delta\text{(}\mathrm{r}\text{-}\mathrm{S}\text{)[}\mathrm{P}_{r}^{\text{(2)}}\mathrm{P}_{\theta}^{\text{(2)}}+\mathrm{P}_{z}^{\text{(2)}}\text{]}$ (7)

The second-order nonlinearity of WS_2_ is ^6^$\chi_{\mathrm{WS}_{2}}^{(2)}=\chi_{xxx}^{(2)}=-\chi_{xyy}^{(2)}=-\chi_{yyx}^{(2)}=-\chi_{yxy}^{(2)}$, where *x* and *y* are the crystal coordinates; *x* is along the armchair direction, which is at an angle of 30° with respect to the zigzag direction. In the WOFN structure, we suppose that the crystallographic orientation of the WS_2_ strip is along the axial direction of the OFN, consistent with the sample fabrication. The nonlinear polarization for the WOFN can be expressed as:

$\begin{matrix} P_{r}^{(2s)}=0 \\ P_{\theta}^{(2s)}=-2\varepsilon_{0}\chi_{_{WS_{2}}}^{(2)}E_{\text{1, }\theta}E_{\text{1, }z}e_{\theta} \\ P_{\text{z}}^{(2)}=\varepsilon_{0}\chi_{_{WS_{2}}}^{(2)}(E_{\text{1, }z}E_{\text{1, }z}-E_{\text{1, }\theta}E_{\text{1, }\theta})e_{\text{z}} \end{matrix}$ (8)

where *E*_1_,*_θ_* and *E*_1_, *_z_* are the mode fields for the fundamental wave (FW) of the WOFN. The second-order nonlinearity of silica is neglected as $\text{χ}_{silica}^{(2)}$ is significantly smaller than $\text{χ}_{WS_{2}}^{(2)}$of the WOFN. For a bare OFN structure, the nonlinear polarization can be expressed as ^5^:

$\begin{matrix} P_{\mathrm{r}}^{\text{(2s)}}=\varepsilon_{0}\text{[}\chi_{_{\perp}}^{\text{(2s)}}\mathrm{E}_{\text{1, }\mathrm{r}}\mathrm{E}_{1,\text{ }\mathrm{r}}\text{+}\chi_{_{\perp||}}^{\text{(2s)}}\text{(}\mathrm{E}_{\text{1, }\theta}\mathrm{E}_{1,\text{ }\theta}+\mathrm{E}_{\text{1, }\mathrm{z}}\mathrm{E}_{\text{1, }\mathrm{z}}\text{)]}e_{\mathrm{r}} \\ P_{\theta}^{\text{(2s)}}=2\varepsilon_{0}\chi_{_{||}}^{\text{(2s)}}\mathrm{E}_{\text{1, }\mathrm{r}}\mathrm{E}_{\text{1, }\theta}e_{\theta} \\ P_{\mathrm{z}}^{\text{(2s)}}=2\varepsilon_{0}\chi_{_{||}}^{\text{(2s)}}\mathrm{E}_{1,\text{ }\mathrm{r}}\mathrm{E}_{1,\text{ }\mathrm{z}}e_{\mathrm{z}} \end{matrix}$ (9)

where the second-order nonlinearities ($\chi_{_{\perp}}^{\text{(2s)}}$,$\chi_{_{\perp||}}^{\text{(2s)}}$, and$\chi_{_{||}}^{\text{(2s)}}$) of fused silica are experimentally measured ^5, 7^.

**Figure S9** compares the nonlinear coupling parameters |*ρ*_2_| for different WS_2_ crystal orientations on the WOFN. It shows that, for a large diameter, |*ρ*_2_| for WS_2_ crystal orientations along the tangential direction of the WOFN is slightly larger than that along the axial direction; it is smaller when the diameter is smaller. This can be intuitively explained by the transverse electric field, which is usually larger than the axial electric field in an optical fiber waveguide with a large diameter.





**Fig. S9 Calculated nonlinear coupling parameters of the WOFN.** We compare two typical WS_2_ crystal orientations in the WOFN, along the tangential and axial directions of the OFN


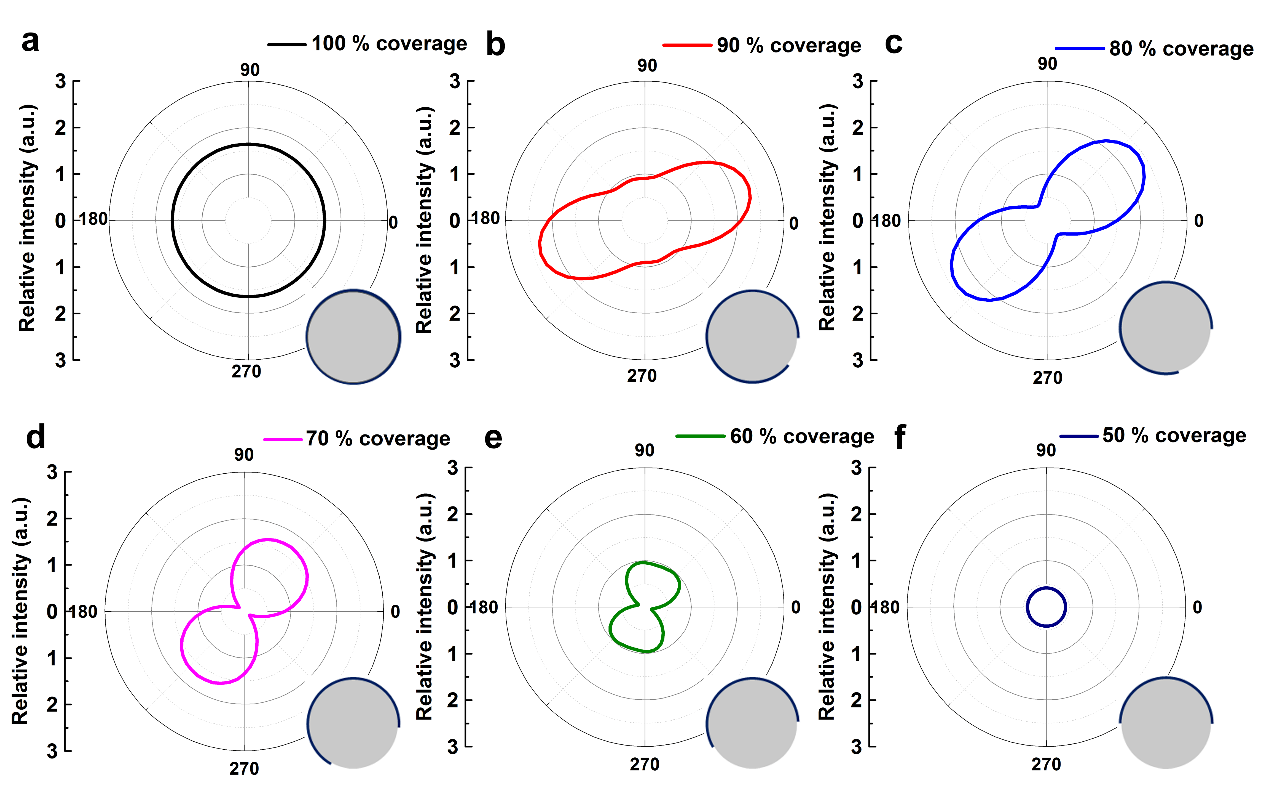


**Fig. S10 Polarization extinction patterns for different coverage ratios of WS_2_ in the WOFN.** The crystal orientation of WS_2_ is along the axial direction of the WOFN. The inset of each panel shows the corresponding WOFN structure with a different coverage of WS_2_

**

**

**Fig. S11 SHG enhancement factor with different coverage ratio of WS_2_.** The diameter of OFN is 800 nm

**
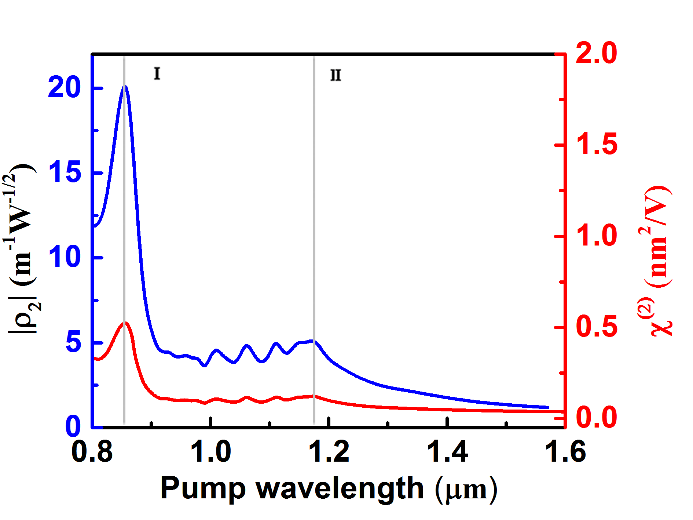
**

**Fig. S12 Calculated nonlinear coupling parameters of the WOFN as a function of the pump light wavelength.** The dispersion of *χ*^(2)^ of WS_2_ is according to the results in Ref. ^6^. The resonance peaks denoted as I and II correspond to a large joint density of states and half of the band-gap energy, respectively

**2.3  Modelling of the strain effect on the SHG in the WOFN**


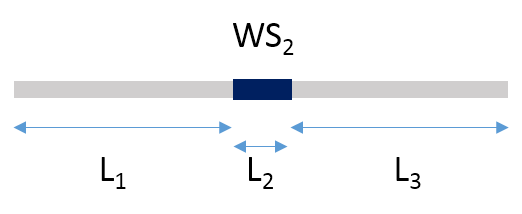


**Fig. S13 Schematic of the WOFN. *L*_1_ and *L*_3_ denote lengths of the OFN, and *L*_2_ is the length of the encapsulating WS_2_ layer**

In our experiments, the waist length of the OFN is usually several millimeters, while the length of the laminating WS_2_ is tens of micrometers. The background SHG from the OFN without WS_2_ interferes with signals from the WS_2_ region, which can be expressed as:

(10)

where *A_L, j_* (*j*=1, 2, 3) is the SHG amplitude in different regions of WOFN (Fig. S13), *β*_2_ is the SHG wavevector, Δ*β* _OFN_, Δ*β* _WOFN_ are the phase mismatch between fundamental and second harmonic waves in OFN and WOFN respectively.

When a strain is applied on the WOFN, the diameter of the OFN decreases, while its length accordingly increases. The photo-elastic effect also contributes to the change of wave-vector in waveguides. These effects contribute to the change in the waveguide dispersion and coupling length, which consequently affect the output SHG intensity. We assumed that the diameter of the OFN and WOFN are uniform in the waist region.





**Fig. S14 Theoretical calculation of the strain tuning of the SHG in the WOFN**

**REFERENCES**

1. Li, Y., et al. Measurement of the optical dielectric function of monolayer transition-metal dichalcogenides: MoS_2_ , MoSe_2_, WS_2_ , and WSe_2_. *Phys. Rev. B* **90**, 205422 (2014).

2. Liu, H. L., Shen, C. C., Su, S. H., and Hsu, C. L. Optical properties of monolayer transition metal dichalcogenides probed by spectroscopic ellipsometry. *Appl. Phys. Lett.* **105**, 201905 (2014).

3. Okamoto, K. *Fundamentals of optical waveguides*, Academic press, 2010.

4. Schell, A. W, Takashima, H., Tran, T. T, Aharonovich, I., Takeuchi, S. Coupling quantum emitters in 2D materials with tapered fibers. *ACS Photonics* **4**, 761-767 (2017).

5. Lægsgaard, J. Theory of surface second-harmonic generation in silica nanowires *J. Opt. Soc. Am. B* **27,** 1317-1324 (2010).

6. Janisch, C., et al. Extraordinary second harmonic generation in tungsten disulfide monolayers. *Sci. Rep.* **4**, 5530 (2014).

7. Rodriguez, F. J., Wang, F. X., and Kauranen M. Calibration of the second-order nonlinear optical susceptibility of surface and bulk of glass. *Opt. Express* **16**, 8704-8710 (2008).
